# Distinct Rumen Microbial Features and Host Metabolic Responses in Three Cervid Species

**DOI:** 10.3390/ani16010116

**Published:** 2025-12-31

**Authors:** Yuhang Zhu, Yunfei Chai, Sibo Chen, Wenxi Qian, Huazhe Si, Zhipeng Li

**Affiliations:** 1 College of Animal Science and Technology, Jilin Agricultural University, Changchun 130118, China; 2 College of Forestry and Grassland Science, Jilin Agricultural University, Changchun 130118, China; 3 College of Animal Science and Technology, Tarim University, Alar 843300, China; 4 Joint International Research Laboratory of Modern Agricultural Technology, Ministry of Education, Jilin Agricultural University, Changchun 130118, China; 5 Key Lab of Animal Production, Product Quality and Security, Ministry of Education, Jilin Agricultural University, Changchun 130118, China

**Keywords:** Cervid, rumen microbiota, fermentation pattern, lipid metabolism

## Abstract

Rumen microbiota is pivotal for nutrient metabolism and physiological adaptation in ruminants. This study investigated the rumen microbial community, fermentation parameters, and serum biochemistry of three Cervid species—Sika deer (*Cervus nippon*), Reindeer (*Rangifer tarandus*), and Milu deer (*Elaphurus davidianus*) (n = 5/group)—fed an identical diet. Using 16S rRNA sequencing and biochemical analyses, we found that while Bacteroidota, Firmicutes, and Proteobacteria were dominant phyla across species. Sika deer and Milu deer exhibited significantly higher microbial diversity and abundance of carbohydrate-digesting genera (e.g., *Butyrivibrio*, *Saccharofermentans*), and pathways of carbohydrate digestion and absorption, starch and sucrose metabolism compared to Reindeer. Conversely, Reindeer showed increased abundances of *Lachnospiraceae ND3007* and butyrate metabolism pathway, and significantly elevated rumen volatile fatty acid concentrations, particularly acetate and butyrate. Serum profiling revealed that Milu deer had significantly higher lipid levels (CHO, TG, LDL-C) but lower total protein and AST levels compared to other species. Notably, WGCNA linked these blood lipid traits to host genes enriched in PI3K-Akt, MAPK, and bile secretion pathways. These findings demonstrate distinct species-specific rumen fermentation patterns and host metabolic adaptations, suggesting a coordinated regulation between the rumen microbiome and host genetics in Cervid.

## Simple Summary

The digestive system of ruminants contains billions of microorganisms that help them digest plants and obtain energy. Different Cervid species have evolved unique ways to adapt to their environments, but how their gut microbes and body metabolism differ remains unclear. In this study, we compared three deer species—Sika deer, Reindeer, and Milu deer—raised on the same diet. We analyzed the rumen microbiota, the fermentation products, and metabolites in serum. We found that although they were fed same diet, rumen microbiota and metabolic profiles were different among three ruminants. Milu deer had higher levels of blood fats, while Reindeer and Sika deer showed higher levels of blood proteins and specific liver enzymes. We also found that the blood genes regulate host metabolism. These findings reveal the specific metabolic adaptations of each species and provide insights for improving their feeding management and conservation strategies.

## 1. Introduction

Ruminants are distinguished from other herbivores by their unique gastric structure, the rumen, which hosts one of the most complex and diverse ecosystems [1]. This microbial ecosystem, composed of bacteria, archaea, protozoa, and fungi, efficiently converts plant fibers and non-protein nitrogen into high-value nutrients such as milk and meat [2]. The fermentation process driven by these microbes produces volatile fatty acids (VFAs) [3], which provide up to 70% of the energy requirements for the host [4]. Among these microorganisms, bacteria are the most abundant and metabolically active, constituting approximately 50% to 70% of the total rumen microbiota [5]. Consequently, the rumen microbiota serves as a critical indicator of rumen health and is intrinsically linked to the nutrient metabolism and physiological well-being of ruminants [1,6]. Growing evidence suggests that rumen microbial composition is shaped by a complex interplay of factors, including age, gender, diet, geographical location, and host genetics [6,7,8,9]. While diet is often considered the primary driver of microbial shifts [7,8,9,10]. While diet is often considered the primary driver of microbial shifts [11], the host’s genetic background plays a pivotal role in selecting and maintaining specific microbial communities. For instance, rumen content exchange studies in beef cattle have shown that the host significantly influences the re-establishment of microbiota after transplantation [12]. Furthermore, recent genomic studies indicate that host genes regulate heritable components of the rumen microbiota by creating unique ecological niches [13,14]. Therefore, exploring the rumen microbiota across different ruminant species is essential for understanding host-specific metabolic adaptations.

Cervidae represents a diverse group of large ruminants inhabiting a wide range of ecological environments, from tropical zones to high-altitude and arctic regions [15]. Different deer species have evolved distinct physiological traits to adapt to their specific niches. For example, Reindeer (*Rangifer tarandus*) possess exceptional lipid storage capabilities and harbor a rumen microbiota highly efficient in fiber digestion and nitrogen recycling to survive in extreme northern environments [16,17]. In contrast, Sika deer (*Cervus nippon*) have been found to host specific tannin-degrading bacteria (e.g., *Streptococcus*) in winter, conferring a stronger ability to utilize tannin-rich forage compared to cattle [18]. However, information regarding the rumen microbiota of Milu deer (*Elaphurus davidianus*) remains limited, with only a few reports identifying specific methanogens such as *Methanocorpusculum* sp. as dominant species [19]. Given these distinct ecological backgrounds, we hypothesize that Reindeer, Sika deer, and Milu deer harbor species-specific rumen microbial profiles that contribute to differences in their fermentation pattern.

Beyond rumen fermentation, blood biochemical parameters reflect the systemic metabolic status of the host. These metabolic profiles are closely tied to species-specific phenological rhythms, particularly antler growth. Unlike other cervids, both male and female Reindeer grow antler [17]. Furthermore, the timing of antler genesis varies significantly among species; for instance, it occurs in winter for Milu deer [20] but in summer for Sika deer [21]. Previous studies have established that circulating hormones (e.g., GH, IGF-1) and metabolites (e.g., triglycerides, cholesterol) are critical regulators of antler development [22,23,24]. Li et al. [25] also showed that rumen microbiota composition shifts across different antler growth stages. However, a comparative analysis integrating rumen microbiota, fermentation parameters, and blood metabolites across these three deer species is lacking. Therefore, the objective of this study is to compare the rumen microbiota and blood biochemical parameters of Reindeer, Milu deer, and Sika deer, aiming to elucidate the species-specific host-microbe interactions and metabolic characteristics that underlie their adaptation and physiological functions.

## 2. Materials and Methods

### 2.1. Animals, Experimental Design, and Sample Collection

This study was conducted using fifteen three-year-old males, comprising five Reindeer, five Milu deer, and five Sika deer. Specifically, the Sika deer, Milu deer, and Reindeer in our study had been maintained at a farm in Changchun for over two years. The feeding trial lasted for eight weeks (September to November). During the initial two weeks, the animals were gradually transitioned from their original diet to a total mixed ration (TMR) of roughage and concentrate (50:50, dry matter basis; Table S1). The animals were fed twice a day (07:00 a.m. and 16:00 p.m.), with Ad libitum access to fresh drinking water. All animal-specific procedures were approved and authorized by the Animal Ethics Committee of Jilin Agricultural University (Approval No. 20220317001).

On 10 November 2022, the animals were anesthetized after morning feed, and then 5 mL of blood was collected through the jugular vein using procoagulant tube. Subsequently, approximately 150 mL of rumen fluid was collected using an oral stomach tube and the first 50 mL was discarded to minimize saliva contamination. The pH of the remaining rumen fluid was immediately measured using a portable pH meter. The samples were then snap-frozen in liquid nitrogen for downstream analysis.

### 2.2. Measurement of Serum Biochemical Parameters and Rumen VFAs

Bloods were centrifuged at 3000× *g* for 15 min at 4 °C to obtain serum. The serum concentrations of glucose (GLU), triglycerides (TG), total cholesterol (TC), high-density lipoprotein cholesterol (HDL-C), low-density lipoprotein cholesterol (LDL-C), aspartate aminotransferase (AST), alanine aminotransferase (ALT), alkaline phosphatase (ALP), total protein (TP), and albumin (ALB) were quantified using commercial kits (Jiancheng Bioengineering Institute, Nanjing, China) with an automatic biochemical analyzer (Mindray, Shenzhen, China). For volatile fatty acid (VFA) analysis, rumen fluids were centrifuged at 10,000× *g* for 10 min at 4 °C. The supernatant was analyzed using gas chromatography (6890GC, Agilent Technologies, Santa Clara, CA, USA) equipped with a flame ionization detector (FID) and a DB-FFAP column according to previous method [26].

### 2.3. DNA Extraction, Sequencing, and Bioinformatics Analysis

Total microbial DNA was extracted from rumen fluids using the MoBio Power Fecal DNA Isolation Kit (QIAGEN, Valencia, CA, USA) following the manufacturer’s protocol. The V3–V4 regions of the bacterial 16S rRNA gene were amplified using primers 341F (5′-CCTACGGGAGGCAGCAG-3′) and 806R (5′-GGACTACHVGGGTWTCTAAT-3′). The resulting amplicons were purified using the QIAquick PCR Purification Kit (QIAGEN, Valencia, CA, USA) and sequenced on the Illumina NovaSeq 6000 platform to generate paired-end reads. Raw paired-end reads were merged using FLASH [27] and subsequently processed using the QIIME 2 [28]. The DADA2 plugin was employed to denoise sequences and generate amplicon sequence variants (ASVs) [29]. Taxonomy was assigned to representative ASVs against the SILVA database (release 138.1) [30]. To normalize sequencing depth, each sample was rarefied to a minimum depth of 19,228 reads. Alpha diversity indices were calculated to assess within-sample diversity. Beta diversity was evaluated using principal coordinate analysis (PCoA) based on Bray–Curtis dissimilarity, as well as Unweighted and Weighted UniFrac distance matrices. Analysis of Similarities (ANOSIM) was performed to test for significant differences in microbial community structure among groups, while the Adonis test was used to quantify the variation explained by grouping factors (999 permutations). Tax4Fun2 was used to predict the functional profiles of the rumen microbiota based on SILVA database (v132) [31].

### 2.4. Network and Correlation Analysis

Visual association networks between differential bacterial genera and fermentation parameters were constructed using the Fruchterman-Reingold layout algorithm in Gephi (v0.10.1) [32]. Additionally, we performed a weighted gene co-expression network analysis (WGCNA) [33]. This analysis integrated the serum biochemical indices obtained in the current study with differentially expressed genes (DEGs) identified in the blood transcriptome of the same species from our previous study (BioProject PRJNA1373567) [34]. Correlations were assessed using the Spearman coefficient. Functional enrichment analysis of gene modules, including Gene Ontology (GO) and Kyoto Encyclopedia of Genes and Genomes (KEGG) pathways, was performed using KOBAS [35], with *p*-values adjusted using the Benjamini–Hochberg method.

### 2.5. Statistical Analysis

All statistical analyses were performed using R software (v4.3.1). The Kruskal–Wallis test was used to assess overall differences among the three groups. Where significant differences were detected, post hoc pairwise comparisons were conducted using the Wilcoxon rank-sum test. *p*-values were adjusted for multiple comparisons using the Benjamini–Hochberg false discovery rate (FDR) method. A *p*-value < 0.05 was considered statistically significant. Data are expressed as the mean ± standard error of the mean (SEM).

## 3. Results

### 3.1. Comparison of Rumen Microbiota and Potential Functions Among Sika Deer, Reindeer, and Milu Deer

**Figure 1:**
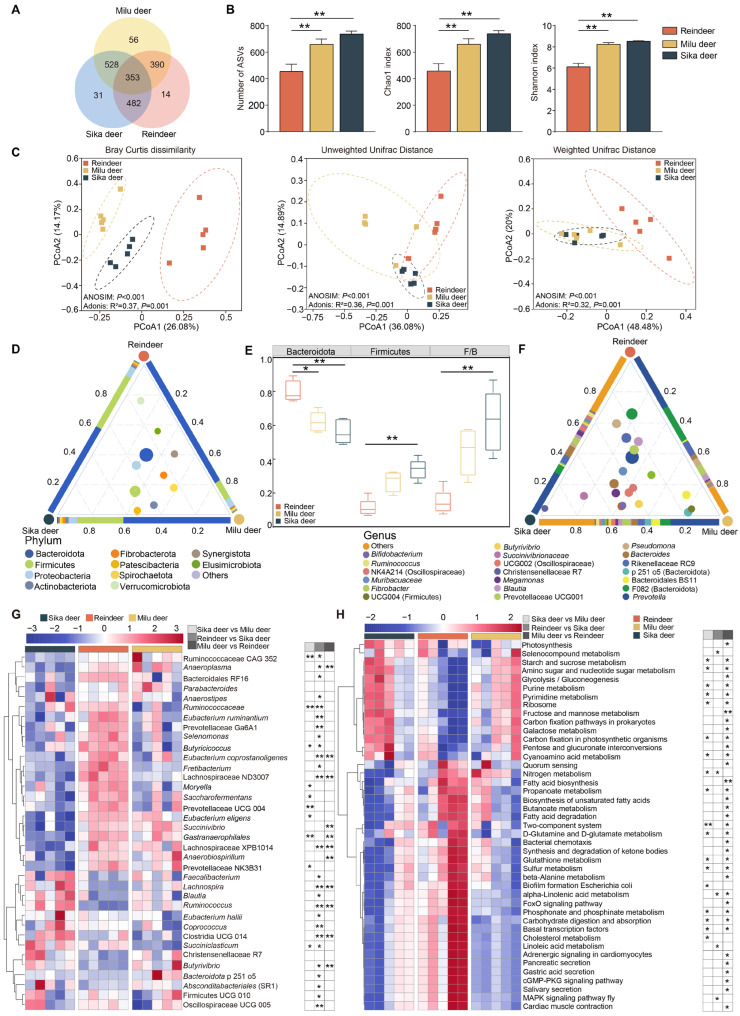
Rumen bacterial community composition and predicted functional profiles. (**A**) Venn diagram showing shared and unique bacterial ASVs among Sika deer, Reindeer, and Milu deer. (**B**) Comparison of alpha diversity indices of the rumen microbiota among the three species. (**C**) PCoA plots based on Bray–Curtis dissimilarity, unweighted UniFrac, and weighted UniFrac distances at the ASV level. The colored dashed ellipses indicate the 95% confidence intervals for each group. (**D**) Ternary plot displaying the average relative abundance of the top ten bacterial phyla. The three vertices represent the three species. Inner circles represent bacterial phyla, with size proportional to abundance. Proximity to a vertex indicates higher abundance of that phylum in the corresponding species. The colors of three triangle sides and inner circles represent different bacterial phyla as defined in the legend. (**E**) Comparison of the relative abundances of Bacteroidota and Firmicutes, and F/B ratio among the three species. (**F**) Average relative abundance of the top twenty bacterial genera among the three species. The colors of three triangle sides and inner circles represent different bacterial genera as defined in the legend. (**G**) Heatmap showing differentially abundant genera among the three species. The color scale represents normalized relative abundance, ranging from low (blue) to high (red). (**H**) Heatmap showing differentially enriched KEGG pathways (Level 3) among the three species. * *p* < 0.05, ** *p* < 0.01.

The rumen bacterial communities were characterized based on 16S rRNA gene sequencing, yielding a total of 1617 ASVs from a sequencing depth ranging from 72,522 to 84,986 reads per sample. Venn diagram analysis revealed that 353 ASVs were shared among the three cervid species, while 14, 56, and 31 ASVs were unique to Reindeer, Milu deer, and Sika deer, respectively (Figure 1A). Alpha diversity analysis indicated that the number of observed ASVs, Chao1 index, and Shannon index were significantly higher in Sika deer and Milu deer compared to Reindeer (Figure 1B, *p* < 0.01). Furthermore, PCoA based on Bray–Curtis dissimilarity, unweighted UniFrac, and weighted UniFrac distances demonstrated distinct clustering, indicating significant differences in microbial community structure among the three species (Figure 1C).

Taxonomic classification identified 15 phyla across all samples (Figure 1D). Bacteroidota was the predominant phylum (Sika deer = 56.1 ± 3.3%, Reindeer = 80.1 ± 2.8%, Milu deer = 62.0 ± 2.7%), followed by Firmicutes (Sika deer = 33.9 ± 2.7%, Reindeer = 11.2 ± 2.3%, Milu deer = 26.8 ± 2.8%) and Proteobacteria (Sika deer = 6.0 ± 1.4%, Reindeer = 5.5 ± 2.0%, Milu deer = 4.7 ± 0.3%). Comparative analysis revealed that the relative abundance of Bacteroidota was significantly higher in Reindeer than in Milu deer (*p* < 0.05) and Sika deer (*p* < 0.01). Conversely, both the relative abundance of Firmicutes and the Firmicutes/Bacteroidota (F/B) ratio were significantly lower in Reindeer compared to Sika deer (*p* < 0.01, Figure 1E). At the genus level, 160 genera were identified (Figure 1F). *Prevotella* was the most abundant genus across all groups (Sika deer = 12.1 ± 3.2%, Reindeer = 24.5 ± 8.2%, Milu deer = 21.4 ± 8.4%), followed by *Bacteroidales F082* (Sika deer = 5.2 ± 0.9%, Reindeer = 21.1 ± 6.0%, Milu deer = 5.7 ± 0.7%). Notably, *Bacteroides* (6.0 ± 1.4%) was prevalent in Sika deer, while *Bacteroidales BS11* (5.0 ± 2.8%) was enriched in Milu deer. In Reindeer, *Rikenellaceae RC9* (8.5 ± 1.9%) and *Bacteroidales RF16* (5.9 ± 0.5%) showed high relative abundances.

Differential abundance analysis identified 36 genera that varied significantly among the three species (*p* < 0.05, Figure 1G). The relative abundances of *Ruminococcus*, *Butyrivibrio*, *Lachnospira*, *Absconditabacteriales (SR1)*, *Eubacterium coprostanoligenes*, *Lachnospiraceae XPB1014*, and *Saccharofermentans* were significantly lower in Reindeer compared to Milu deer and Sika deer (*p* < 0.05). In contrast, *Fretibacterium*, *Lachnospiraceae ND3007*, *Bacteroidales RF16*, and *Moryella* were significantly enriched in Reindeer compared to the other groups. Functional prediction analysis using Tax4Fun2 revealed significant differences in 43 metabolic pathways among the three groups (*p* < 0.05, Figure 1H). Specifically, pathways related to starch and sucrose metabolism, amino sugar and nucleotide sugar metabolism, carbohydrate digestion and absorption, purine metabolism, and pyrimidine metabolism were significantly less abundant in Reindeer compared to Milu deer and Sika deer. Conversely, pathways associated with propanoate metabolism, butanoate metabolism, signal transduction, and sulfur metabolism were significantly enriched in Reindeer.

### 3.2. Comparison of Rumen Fermentation Parameters Among the Three Cervid Species

**Figure 2:**
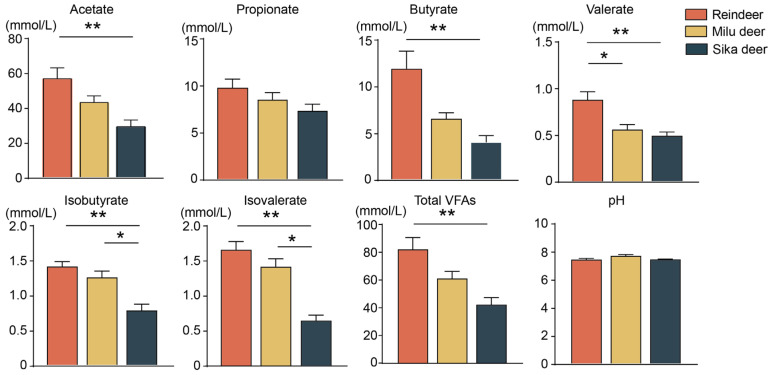
Comparison of fermentation parameters in the rumen fluid among Reindeer, Milu deer, and Sika deer. * *p* < 0.05, ** *p* < 0.01.

Analysis of rumen fermentation parameters revealed distinct VFA profiles among the species (Figure 2). Specifically, the concentrations of acetate, butyrate, isobutyrate, valerate, isovalerate, and total VFAs were significantly higher in Reindeer compared to Sika deer (*p* < 0.01). Furthermore, Reindeer exhibited significantly higher valerate concentrations than Milu deer (*p* < 0.05). Comparisons between Milu deer and Sika deer showed that the concentrations of isobutyrate and isovalerate were significantly higher in Milu deer. In contrast, no significant differences were observed in rumen pH values among the three species.

### 3.3. Correlation Analysis Between Rumen Microbiota and Fermentation Parameters

**Figure 3:**
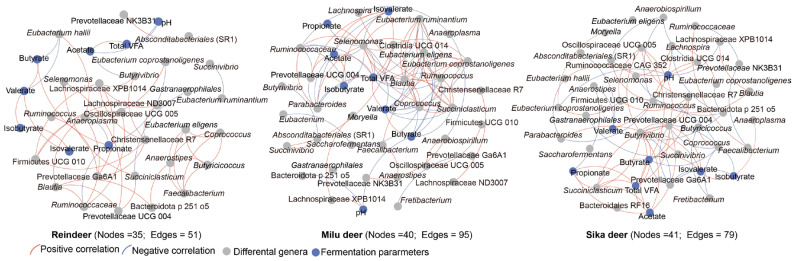
Association networks between fermentation parameters and differentially abundant genera in Sika deer, Reindeer, and Milu deer. Circular nodes represent fermentation parameters (blue) and bacterial genera (gray). Edges denote correlations between nodes, with red lines indicating positive correlations and blue lines indicating negative correlations.

Network analysis was performed to evaluate the associations between differential bacterial genera and fermentation parameters (Figure 3). The resulting correlation networks exhibited distinct topological structures for each species: Sika deer (41 nodes, 79 edges), Reindeer (35 nodes, 51 edges), and Milu deer (40 nodes, 95 edges). Notably, the Milu deer network displayed the highest average degree of connectivity, whereas the Reindeer network was the least complex.

In Sika deer, a stable and densely connected module of positive correlations was identified, comprising *Butyrivibrio*, *Succiniclasticum*, *Prevotellaceae UCG-004*, *Christensenellaceae R7*, *Oscillospiraceae UCG-005*, and key fermentation parameters (total VFA, acetate, butyrate, and isovalerate). For Reindeer, positive correlations were primarily observed among *Christensenellaceae R7*, *Oscillospiraceae UCG-005*, *Lachnospiraceae XPB1014*, and *Anaeroplasma*. In the Milu deer network, a robust correlation module was observed linking *Ruminococcus*, *Clostridia UCG-014*, *Coprococcus*, *Selenomonas*, *Eubacterium ruminantium*, *Eubacterium eligens*, and total VFA (Figure 3).

### 3.4. Comparison of Serum Biochemical Parameters Among the Three Cervid Species

**Figure 4:**
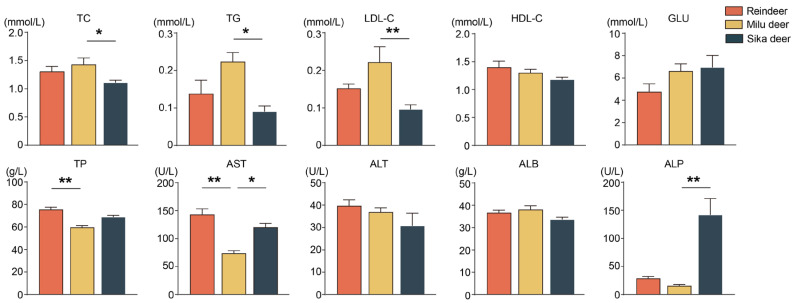
Comparison of serum biochemical parameters among Reindeer, Milu deer, and Sika deer. * *p* < 0.05, ** *p* < 0.01.

Analysis of serum biochemical profiles revealed significant interspecies differences (Figure 4). Specifically, serum concentrations of total cholesterol (TC), triglycerides (TG), and low-density lipoprotein cholesterol (LDL-C) were significantly higher in Milu deer compared to Sika deer (*p* < 0.05). Reindeer exhibited significantly higher levels of aspartate aminotransferase (AST) and total protein (TP) compared to Milu deer (*p* < 0.01). Furthermore, serum AST and alkaline phosphatase (ALP) levels were significantly elevated in Sika deer compared to Milu deer (*p* < 0.05).

### 3.5. WGCNA Between Serum Biochemical Parameters and DEGs in Blood

**Figure 5:**
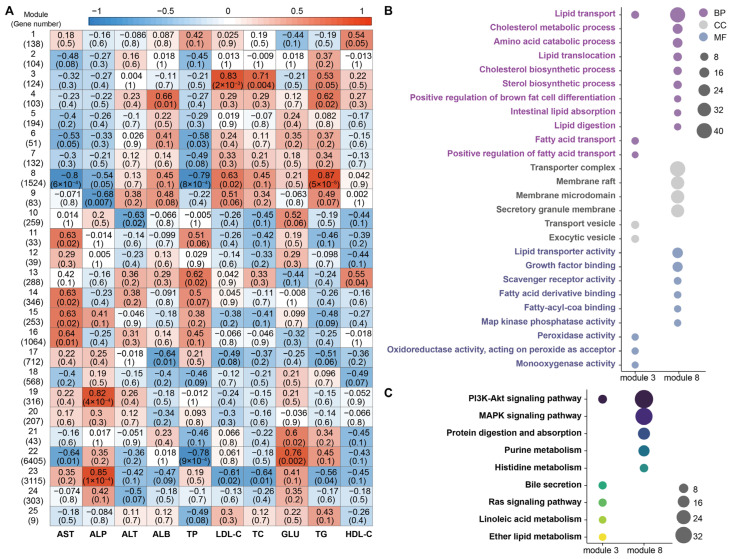
WGCNA identifying associations between blood DEGs and serum biochemical parameters. (**A**) Heatmap displaying correlation coefficients between 25 co-expression modules (rows) and 13 serum biochemical parameters (columns). In each cell, the upper value represents the correlation coefficient, and the lower value represents the corresponding significance level (*p*-value). The color gradient ranges from blue (negative correlation) to red (positive correlation), with intensity reflecting the strength of the correlation. (**B**) GO enrichment analysis of genes within Modules 3 and 8. Dot size is proportional to the number of genes annotated to each GO term, while dot color denotes the GO category: Biological Process (BP, purple), Cellular Component (CC, grey), and Molecular Function (MF, blue). (**C**) KEGG pathway enrichment analysis for Modules 3 and 8. Dot size reflects the number of genes enriched in each pathway. Dot color indicates statistical significance, ranging from yellow (lower significance) to dark purple (higher significance).

In our previous study, we investigated distinct blood gene expression profiles among Sika deer, Reindeer, and Milu deer [34]. Building on this, we performed WGCNA to integrate the current serum biochemical parameters with the previously identified blood differentially expressed genes (DEGs). This analysis identified 25 co-expression modules (Figure 5A). We observed significant positive correlations between lipid metabolism-related parameters (specifically LDL-C and TC) and Modules 3 and 8. Module 3, which contains genes such as *ERFE*, *CYP4F2*, *PLA2G1B*, and *BMP4*, showed significant positive correlations with LDL-C, TG, and TC (*p* < 0.05). Similarly, Module 8 (containing *MAPK14*, *METRNL*, and *PTGS2*) was positively correlated with TG and LDL-C (*p* < 0.05).

Subsequent GO and KEGG pathway enrichment analyses were conducted on these two key modules. For Module 3, DEGs were primarily enriched in biological processes related to lipid and fatty acid transport; cellular components including transport vesicles and exocytic vesicles; and molecular functions such as peroxidase and monooxygenase activities. For Module 8, DEGs were significantly enriched in biological processes, including lipid transport and the positive regulation of brown fat cell differentiation. Enriched cellular components for Module 8 included transporter complexes and membrane rafts, while molecular functions involved lipid transporter activity and fatty acid derivative binding (Figure 5B). KEGG pathway analysis revealed that DEGs in Module 3 were mainly associated with the PI3K-Akt signaling pathway, bile secretion, and the Ras signaling pathway. DEGs in Module 8 were enriched in pathways including PI3K-Akt signaling, MAPK signaling, and protein digestion and absorption (Figure 5C).

## 4. Discussion

Our results demonstrated that Bacteroidota, Firmicutes, and Proteobacteria were the dominant phyla in the rumen of Sika deer, Reindeer, and Milu deer, consistent with previous findings in other cervid species, including roe deer, Sika deer, and red deer [9,26,36]. At the genus level, *Prevotella* and *Bacteroidales F082* were the most dominant taxa across all three species. *Prevotella* has been identified as a core genus in the rumen of numerous cervids, such as Tarim red deer, moose, and Sika deer [37,38,39]. This genus is capable of degrading various polysaccharides to generate propionate, an essential substrate for hepatic gluconeogenesis [40]. Previous studies have indicated that *Bacteroidales F082* likely plays a role similar to that of *Bacteroides* in carbohydrate decomposition and organic matter fermentation, possibly contributing to propionate production [36,41]. These findings highlight the fundamental roles of these microorganisms in the rumen function of Cervidae.

Despite identical dietary conditions, we observed significant differences in the abundance, diversity, and structure of the rumen microbiota among Sika deer, Reindeer, and Milu deer. This is consistent with our previous observation that the rumen microbiota of hybrid offspring (Sika deer × Red deer) remained highly similar to that of their parents [7], suggesting that the structure and composition of the rumen microbiota are strongly influenced by the host genetic background. Notably, the F/B ratio was significantly lower in Reindeer compared to Sika deer and Milu deer. It has been demonstrated that a decrease in Firmicutes or an increase in Bacteroidota is associated with increased total VFA concentrations [42], which indicates enhanced fermentation efficiency and energy supply to host [43]. Accordingly, the concentrations of total VFAs in the rumen of Reindeer were higher than those in Milu deer and Sika deer. Given that alterations in the metabolic activity of the Reindeer digestive tract may constitute a compensatory mechanism for energy and nutrient expenditure [44], these results suggest that the rumen microbiota contributes significantly to the adaptation of Reindeer to the Arctic environment.

Comparative analysis revealed that the relative abundances of *Bacteroidales RF16* and *Lachnospiraceae ND3007*, as well as pathways related to butyrate metabolism, were elevated in the rumen of Reindeer. A significant decrease in *Bacteroidales RF16* has previously been observed in the gastrointestinal tract of diarrheal Milu deer compared to healthy individuals [45], suggesting a role for this taxon in maintaining gastrointestinal health. *Lachnospira*, belonging to the family *Lachnospiraceae*, is widely reported in the cervid digestive tract and is involved in fiber degradation and butyrate production [46,47,48,49]. Butyrate produced by the gut microbiota is involved in regulating bone mineral density, particularly in response to exercise [50]. Notably, our previous study demonstrated that butyrate-producing fermentation was significantly upregulated during the rapid antler growth phase of Sika deer [51]. Given the elevated abundance of butyrate-producing bacteria and increased butyrate metabolism in rumen of Reindeer, it is plausible that the rumen microbiota promotes antler development by modulating host metabolic and physiological pathways.

Conversely, *Saccharofermentans*, *Butyrivibrio*, and *Parabacteroides* were significantly more abundant in the rumen of Sika deer and Milu deer relative to Reindeer. *Saccharofermentans* plays a crucial role in carbohydrate fermentation, generating fumarate, lactate, and acetate [52]. Members of *Parabacteroides* possess polysaccharide utilization loci that facilitate carbohydrate utilization with the production of acetate, propionate, and butyrate [53]. *Butyrivibrio* is known to degrade hemicellulose, participate in protein breakdown, and facilitate the biohydrogenation of fatty acids [54,55,56]. Functional prediction analysis indicated that pathways related to starch and sucrose metabolism, carbohydrate digestion and absorption, and amino sugar and nucleotide sugar metabolism were enriched in the rumen of Sika deer and Milu deer. These findings indicate that the composition of the rumen microbial community plays a critical role in determining fermentation patterns.

Network analysis revealed distinct interaction patterns among the species. The Reindeer microbiome exhibited higher degree of positive correlations, whereas Sika deer and Milu deer networks showed higher degree of negative correlations. Recent evidence demonstrates that core taxa produce essential metabolites and encode crucial fiber-degrading enzymes, engaging in cross-feeding to provide non-core microbes with vital nutrients [57]. In dairy cows, strong microbial cooperation has been linked to better adaptability to nutritional changes [58]. Our results suggest potential cooperation among rumen microbiota in Reindeer, contrasting with the competitive dynamics in Sika deer and Milu deer. Furthermore, *Prevotellaceae UCG-004* and *Christensenellaceae R-7* were identified as key taxa in the networks of both Sika deer and Reindeer, while *Eubacterium* was central in Milu deer. *Prevotellaceae UCG-004* contributes to polysaccharide degradation, enhancing rumen fermentation capacity and promoting rumen epithelium development, and is positively correlated with acetate and butyrate concentrations [59,60,61]. The *Christensenellaceae R-7* is important for maintaining gut structure and function and is primarily involved in amino acid, peptide, and lipid metabolism [62]. Members of *Eubacterium*, such as *E. coprostanoligenes*, *E. ruminantium*, and *E. eligens*, are potential probiotics known for cholesterol-lowering effects and cellulose degradation [63,64,65]. These findings reveal that microbial interactions and cross-feeding mechanisms differ among the three cervid species even under the same dietary conditions.

The levels of TC, TG, and LDL-C were significantly higher in Milu deer than in Reindeer and Sika deer. Total cholesterol (TC) is a key product of lipid metabolism, and elevated levels may indicate a lower utilization rate of body fat [66]. LDL-C transports cholesterol from the liver to peripheral tissues, whereas HDL-C facilitates reverse transport [67]. These results suggest a species-specific tendency towards enhanced lipid synthesis and storage in Milu deer. In contrast, serum AST and TP levels were lower in Milu deer compared to the other two species. Increased TP indicates improved absorption and utilization efficiency of amino acids and proteins, as well as enhanced hepatic protein synthesis and metabolic function [68]. ALT is produced by liver and plays an important role in amino acid metabolism [69]. Together, these distinct blood biochemical profiles reflect species-specific adaptations regarding lipid mobilization efficiency and protein metabolic dynamics.

WGCNA revealed significant correlations between lipid metabolism genes (including *ERFE*, *CYP4F2*, *PLA2G1B*, *MAPK14*, *METRNL*, and *PTGS2*) and serum TG and LDL-C levels. *ERFE* has been reported to suppress excessive abdominal fat accumulation and improve glucose tolerance [70]. High expression of *CYP4F2* contributes to the metabolism of arachidonic acid and omega-3 polyunsaturated fatty acids [71]. *PLA2G1B* facilitates dietary fat absorption and can promote diet-induced obesity [72]. *METRNL* is a cold-induced circulating hormone that stimulates energy expenditure and the “browning” of white adipose tissue [73]. Similarly, *PTGS2* (encoding COX-2) is a crucial downstream effector in the induction of beige adipocytes [74], and *MAPK14* (p38 MAPK) serves as a central signaling hub for activating the thermogenic program in response to cold stress [75]. The enrichment of these positive regulators suggests that adult Reindeer may retain the plasticity to recruit thermogenic adipocytes to cope with extreme cold environments. Moreover, the PI3K-Akt and MAPK signaling pathways were enriched in Modules 3 and 8, while bile secretion was enriched in Module 3. Both PI3K-Akt and MAPK signaling pathways have been reported to be involved in antler stem cell proliferation and growth [21,76]. Bile acids play a crucial role in lipid digestion and absorption, and our previous study revealed their role in promoting antler stem cell proliferation [24]. These findings suggest a potential mechanistic connection between lipid metabolism—particularly bile acid-related processes—and antler development.

## 5. Conclusions

Our study reveals significant interspecific divergence in the rumen microbiota, fermentation patterns, and host metabolic profiles among Sika deer, Reindeer, and Milu deer maintained under identical dietary conditions. Specifically, Milu deer exhibit a metabolic signature characterized by enhanced lipid storage, whereas Reindeer and Sika deer display traits indicative of active protein turnover. Furthermore, integrated WGCNA highlights a potential regulatory network linking lipid metabolism genes to key signaling pathways, such as PI3K-Akt and MAPK, which may underlie the observed physiological phenotypes. These findings underscore the distinct metabolic adaptations evolved by these cervid species, providing insights for optimizing species-specific nutritional strategies and advancing our understanding of cervid physiology. Moreover, these findings offer insights for developing nutritional strategies (e.g., supplementing with polysaccharides, oligosaccharides, and amino acids) that could affect the gastrointestinal tract and host metabolism, contributing to the conservation of endangered species such as the Milu deer and the improvement of productivity. In future studies, isolating butyrate-producing microbes and investigating metabolic profiles based on shotgun sequencing and metabolomics would be valuable for understanding the roles of the gastrointestinal microbiome in host adaptation and metabolism, particularly by including a larger sample size.

## Data Availability

Blood bulk RNA-seq data and 16S rRNA sequencing data have been deposited in NCBI under accession numbers PRJNA1373567 and PRJNA1373525, respectively.

## Supplementary Materials

The following supporting information can be downloaded at: https://www.mdpi.com/article/10.3390/ani16010116/s1, Table S1: Ingredients and chemical compositions of the experimental diets.

## Abbreviations

The following abbreviations are used in this manuscript:

TC	total cholesterol
TG	triglyceride
LDL-C	low-density lipoprotein cholesterol
GLU	glucose
HDL-C	high-density lipoprotein cholesterol
AST	aspartate aminotransferase
ALT	aminotransferase
ALP	alkaline phosphatase
TP	total protein
ALB	albumin
PCoA	principal coordinates analyses
ANOSIM	analysis of similarities
DEGs	differently expressed genes
GO	gene ontology
KEGG	kyoto encyclopedia of genes and genomes
SEM	standard error of the mean
VFA	volatile fatty acid
WGCNA	weighted correlation network analysis
