# Supplementary material for: Distinct Rumen Microbial Features and Host Metabolic Responses in Three Cervid Species
Source: Animals (Basel). 2025 Dec 31;16(1):116. doi: 10.3390/ani16010116 (PMC12784906; doi:10.3390/ani16010116)
Supplement: Supplementary file 1 [file animals-16-00116-s001.zip › animals-4069456-supplementary.pdf]

Table S1. Ingredients and chemical compositions of the experimental diets

Ingredient	Proportion（%）	Nutrient levels	Content（%）
Corn	25.00	Dry matte %	92.57
Soybean meal	10.00	Crude protein %	17.68
Corn germ	5.00	Metabolic energy (MJ/kg)	10.78
DDGS	4.50	ether extract %	2.34
Corn germ	4.00	Neutral detergent fiber %	58.94
Corn silage	50.00	Acid detergent fiber %	32.54
NaCl	0.50		
Premix①	1		
Total	100		

DDGS = Distillers Dried Grains with Solubles

① One kilogram premix provided the following salt 3 g, calcium 2.5 g, phosphorus 1.5 g, vitamin A 180 mg, vitamin E 360 mg, vitamin D3 100 mg, Fe 700 mg, Cu 260 mg, Zn 750 mg, Mn 450 mg, Se 110 mg, Co 90 mg.

② nutrient levels were measured values.
